# A Global Examination on the Attitudes Toward Intimate Partner Violence Against Women: A Multilevel Modeling Analysis

**DOI:** 10.1177/10778012251338388

**Published:** 2025-04-30

**Authors:** Yangjin Park, Jingyeong Song, Bethany Wood, Toni Gallegos, Saltanat Childress

**Affiliations:** 1 12329 The University of Texas at Arlington, Arlington, TX, USA; 2 1974 The University of Queensland, QLD, Australia

**Keywords:** intimate partner violence, violence against women, multilevel modeling, the World Values Survey, ecological framework

## Abstract

Intimate partner violence against women (IPVAW) is a global health and human rights issue. This study explores the multilevel risk factors—individual, interpersonal, neighborhood, and national—that influence attitudes toward wife abuse. Using multilevel modeling, data from the World Value Survey and country-level datasets (*n* = 76,025), our findings suggest that improving perceptions of gender inequality (individual-level) and emphasizing the values of democracy (country-level) are particularly critical to reducing positive attitudes toward IPVAW. Additionally, merely enacting domestic violence laws may not change attitudes that tolerate IPVAW. Instead, the proper implementation, resources, and public awareness of these laws are crucial, particularly among women.

## Introduction

Intimate partner violence against women (IPVAW) has drawn international attention as a public health problem (Devries et al., 2013). On a global scale, in 2018, approximately 492 million ever-partnered women between the ages of 15 to 49 were documented to experience intimate partner violence in the form of physical or sexual, or both at least once in their lifetime (Sardinha et al., 2022). Due to the complex and multifaceted nature of intimate partner violence (IPV), IPV has been interchangeably used with various terms such as domestic violence, domestic abuse, family violence, interpersonal violence, and spousal abuse (Beck et al., 2013, Park et al., 2022). Exposure(s) to IPVAW is likely to entail negative physical and emotional impacts, which can either be immediate or last over time (Campbell, 2002). The ecological framework provides a useful theoretical approach to comprehensively understand the multifaceted risk factors associated with IPVAW (Michau et al., 2015). In particular, the bioecological model of human development (Bronfenbrenner & Morris, 2006) describes various domains of experiences that influence human behavior and development. In this framework, a person's environment is a set of interconnected ecological layers (e.g., microsystem and macrosystem). Each layer represents increasingly distant aspects of the environment, which collectively shape an individual's development through ongoing reciprocal interactions. In the context of IPVAW, each domain such as individual, family, and community has its unique contribution to IPVAW (Smith Slep et al., 2014). Multilevel modeling (MLM) has been a promising methodological approach for applying the ecological perspective on research for IPVAW because it allows us to assess the various levels of analysis (Javdani et al., 2011; Raudenbush & Bryk, 2002). To date, a substantial body of research has applied MLM to examine various factors associated with IPVAW at the country level (Marshall et al., 2011; Koenig et al., 2006; Yoshihama & Bybee, 2011), the regional level (Uthman et al., 2009), and the international level (Hayes & Pinchevsky, 2023).

However, there are a few limitations in the current literature. First, a paucity of research exists in examining the various layers of risk factors to understand attitudes toward wife abuse on a global scale. Despite an awareness of the importance of the protective sphere of the macro-level domain, societal-level factors such as democracy, domestic violence law, and civil liberties on attitudes toward IPVAW have been understudied. Second, given that the victims of IPVAW are women, it is important to understand how the effect of sex on attitudes toward wife abuse varies by country. Third, despite the significance of the issue of IPVAW, prior research shows that one of the challenges in collecting data on interpersonal violence is difficulty in eliciting responses due to fear, stigma, shame, and different definitions of violence (Diamond-Smith & Rudolph, 2018). To address this, a body of studies circumvented the challenges of gathering information on violence by asking about attitudes toward the justifiability of interpersonal violence (Alvarez et al., 2015; Forbes et al., 2005; Simpson et al., 2018; Abani & Pourmehdi, 2021, Park et al., 2023). Particularly, a woman who justifies IPVAW is less likely to leave an abusive husband or seek help (Dasgupta, 2019; Haushofer et al., 2019). Similarly, a man who justifies IPVAW may be more likely to perpetrate violence against a woman. Furthermore, according to Khan and Islam (2018) and Dhar et al. (2019), discriminatory gender attitudes impact a range of outcomes such as income, work status, autonomy, usage of health services, and education. Hence, to overcome these limitations in MLM and IPVAW, this study examines individual, interpersonal, neighborhood, and national levels of risk factors that are associated with justifying wife abuse on a global scale. This study further examines the role of sex in the association between ecological risk factors and attitudes toward wife abuse.

### Individual Domain

#### Life satisfaction and economic hardship

Life satisfaction and financial satisfaction are critical precursors of violent behaviors. A body of research has consistently found a negative association between life satisfaction and violence (MacDonald et al., 2005; Hamdan-Mansour et al., 2012; Ramirez & Andreu, 2006). Aggressive behaviors generally decrease as life satisfaction increases (Ronen et al., 2013). In the context of IPVAW, women with low life satisfaction are more likely to report experiencing abuse from their spouses (Hammet et al., 2020; Hussain et al., 2020). Furthermore, economic hardship, inclusive of poverty, unemployment, and food insecurity, is associated with higher rates of IPVAW (Ahmadabadi et al., 2020). Jewkes (2002) explains that economic hardship can contribute to frustration, stress, and feelings of powerlessness among men, increasing the risk of IPVAW. In support of this position, Ahmadabadi and colleagues (2020) demonstrated that when a husband earns a low income, wives experience a higher rate of intimate partner violence. Furthermore, economic hardship may prevent women from escaping abusive partners. Low-income women experiencing IPVAW often lack the resources and access to civil and legal representation necessary to leave abusive partnerships (Teufel et al., 2021; National Network to End Domestic Violence, 2017). A growing body of global research demonstrates that cash transfers and other poverty-reduction initiatives can decrease IPVAW (Barrington et al., 2022; Spencer et al., 2020; Buller et al., 2018).

### Interpersonal Domain

#### Lack of trust

Lack of trust in the intimate partner relationship can lead to physical and emotional violence perpetration (Paphitis et al., 2022; Toplu-Demirtaş et al., 2022). According to Bowlby (1982), adults’ attachment is connected to threatening or unsafe experiences that the individual faced during infancy and if their parents were present at that time. Consequently, those who have high levels of attachment anxiety often have negative self-views and are guarded about being close to others in romantic relationships (Cope et al., 2024; Rodriguez et al., 2015). An individual with attachment anxiety shows excessive concerns, fear of abandonment, constant rumination about the relationship, and rejection issues resulting in distrust in relationships (Civilotti et al., 2021). For men, previous experiences from childhood or relationships can impact how they view or approach current partners. If they had experiences of distrust or betrayal, it could lead to continued feelings in the new relationship and ultimately lead to the perpetration of violence toward women (Augusta-Scott, 2020; Rodriguez et al., 2015). Meanwhile, Copp and colleagues (2017) argue that women's distrust in men has not appeared to stop them from dating, due to the hope of finding the “right man.” Consequently, some women may fall back into the cycle of abuse as they sometimes without self-awareness move from one abusive relationship to another (Guyon et al., 2021). This vicious cycle of lack of trust may lead some women to hold onto an attitude of accepting violence from their partners.

#### Gender inequality

Gender inequality is a key component of IPVAW, and women may experience this injustice throughout the life course (Sánchez-Prada et al., 2023). Studies demonstrate that gender is closely related to instances of IPV, particularly highlighting that men perpetrate well over most of the violence against women (Metheny & Stephenson, 2021; Kearns et al., 2020). Gender in a societal context is viewed and practiced with the belief that men ultimately hold power over women despite the progress of women's rights in some areas (Karlsson et al., 2022; Serrano-Montilla et al., 2020). These societal views contribute to the acceptance of IPVAW by reinforcing the expectation that women should hold less power than men (McCarthy et al., 2018). This perspective may be amplified when traditional gender roles are held in high regard at the macro and micro levels since it further takes the power away from women (Sánchez-Prada et al., 2023).

### Neighborhood Domain

Women living in poverty with less education tend to be concentrated in disadvantaged neighborhoods throughout the globe (Iceland & Scopilliti, 2008; Massey et al., 2009). Disadvantaged neighborhoods, inclusive of high rates of community poverty, unemployment, less educated residents, and physical disorder (Pinchevsky & Wright, 2012), are positively associated with higher prevalence of IPVAW regardless of other individual or community factors (Beyer et al., 2015; Pinchevsky & Wright, 2012; Voith, 2019). Furthermore, women who remain in disadvantaged neighborhoods may be more at risk of long-term IPVAW. Longitudinal analysis demonstrates that a high risk for IPVAW can become chronic in underprivileged neighborhoods, suggesting that this community-level disadvantage may amplify or reproduce IPVAW over time (Gracia et al., 2021).

### National Domain

Individual nations have the power to influence and deter structural violence against women through policy options (Caprioli, 2004). For example, empirical research shows that low government spending on social services and social welfare (e.g., education, safety, and health) are critical risk factors for femicide (Palma-Solis et al., 2008). Furthermore, accessible divorce laws are found to reduce abuse through two possible channels: by enabling women to escape abusive relationships and by enhancing their bargaining power (Brassiolo, 2016). In this study, three aspects of the national domain of risks, namely, democracy, domestic violence law, and civil liberties on IPVAW, are highlighted in the literature.

#### Democracy

The value of democracy supports human rights and peace while deterring aggression and violence (U.S. Department of State, 2024). As the inherent characteristic of democracy seeks to exclude violence, individuals in a democratic society are cultivated and encouraged to express their views and thoughts through a rational deliberation process (Schwarzmantel, 2011). Democracy is important in reducing the frequency and severity of human rights violations, including violence (De Mesquita et al., 2005). In analyzing 95 countries, Asal and Brown (2010) found that the higher value placed on democracy by citizens tended to decrease interpersonal violence. In contrast, lack of democracy increases the vulnerability to violence and decreases security for women (Nikolic-Ristanovic, 1999).

#### Domestic violence law

Weak enforcement of rights and a legal system that tolerates IPVAW may lead to a higher level of condoning IPVAW (Barajas-Sandoval et al., 2023). Ortiz-Barreda and Vives-Cases (2013) identified 124 countries having some types of legislation regarding violence against women. Furthermore, in countries where gun ownership is allowed, studies indicate that laws preventing IPV batterers from accessing firearms explain the decline in intimate partner homicide (Zeoli et al., 2020). Exposing private violence into the public area is not just about increasing the involvement of criminal justice agencies but also ensuring the victims’ effective support that moves toward a violence-free future (Burton, 2009). A national-level effort to reduce IPV through legislation may be most salient in the form of protection orders to serve as an expression of public policy that IPV is intolerable (Stoever, 2014). Although many countries have enacted laws to address violence, women in countries like India are not fully protected by the law, increasing the risk of being exposed to IPVAW (Subramaniam & Krishnan, 2016).

#### Restricted civil liberties

Restricted civil liberties refer to discriminatory social institutions that include both formal and informal social norms, practices, and laws, which limit women's political rights and fundamental liberties to participate in raising political voice, freedom of movement and access to justice, and citizenship rights (Organisation for Economic Co-operation and Development [OECD], 2024). Htun and Weldon (2012) conducted a four-decade longitudinal study of 70 countries and found that autonomous social movements brought a lasting impact on policies addressing violence against women by implementing feminist principles in both national and international institutions. Legal representations can significantly impact the quality of life of IPV victims through protective orders, improved safety, and financial self-sufficiency (Lee & Backes, 2018). However, without proper civil liberties, women are likely to be exposed to higher levels of IPV without proper institutional protections.

### Sex Differences in Country-Level Factors

The influence of sex on attitudes toward wife abuse might differ depending on country-level factors. Considering that the primary victims of IPVAW are women, country-level factors may have different effects on sex. A general notion of security is postulated to be sex neutral, with the presumption that men and women share an identical viewpoint. However, Caprioli (2004) argues that democracy and human rights may be viewed and impacted differently by each sex. If women are not explicitly recognized as the source for complaints and survival of violence in the legal text, legislation may be manipulated against women (Ortiz-Barreda & Vives-Cases, 2013; Pan American Health Organization, 2004; United Nations Entity for Gender Equality and the Empowerment of Women, 2011). Consequently, the absence or lack of country-level protection may increase the risk of IPVAW.

### The Current Study

To our knowledge, despite the global awareness of the urgency of IPVAW (Devries et al., 2013), little research has examined ecological risk factors associated with the attitudes toward IPVAW on a global scale. Hence, building upon previous research, this study examined risk factors in individual (i.e., life and financial satisfaction), interpersonal (i.e., lack of trust and gender inequality), neighborhood, and national (i.e., democracy, domestic violence law, and civil liberties) domains on the attitudes toward IPVAW considering the sex differences. Although conceptually each domain is distinctive, our study focuses on how they are experienced, such as individually experienced (level-1 risk) and nationally structured (level-2 risk). Therefore, our study included individual, interpersonal, and neighborhood domains as level-1 risk factors and the national domain as level-2 factors.

The following three hypotheses are examined using multilevel modeling: 1) life and financial satisfaction, lack of trust, gender inequality, and neighborhood disadvantage (level-1 risk factors) are positively associated with the attitudes toward IPVAW; 2) lack of democracy, domestic violence law, and civil liberties (level 2 risk factors) are positively associated with the attitudes toward IPVAW; and 3) sex moderates the association between level-2 risk factors and the attitudes toward IPVAW.

## Methods

### Data Source and Participants

To comprehensively analyze the research question of our study, this study integrated multiple sources of datasets from the World Value Survey (WVS), Economist Intelligence Unit's Democracy Index (EIU), and OECD to create a robust master dataset. The WVS is a public secondary dataset that evaluates the influence of human values, beliefs, and societal changes on political, economic, and social attitudes across 88 countries and territories (Haerpfer et al., 2021). As a time-series dataset, the WVS spans multiple waves, from wave 1 (1981–1984) to wave 7 (2017–2021), with data collected in five-year intervals. The sample design is structured to capture a full probability sample of residents aged 18 years and older in 88 countries. In some countries, full probability sampling was not feasible due to high costs. Instead, a nationally representative sample was collected using multi-stage territorial-stratified selections and random route sampling. Interviews were primarily conducted face-to-face at the respondent's residence, either through a paper questionnaire or a computer-assisted personal interview. To maintain cross-national comparability, the same master questionnaire (in English) was used in all participating countries. Further details on the WVS can be found in the following link (www.worldvaluessurvey.org). To examine the latest global trends in individuals’ attitudes toward wife abuse, this study utilized the most recent wave of data (Wave 7; 2017–2021).

Country-level data were sourced from three additional sources. Data on democracy were obtained from the EIU's Democracy Index from 2016 (EIU digital solutions, n.d.). The data for Restricted Civil Liberties were derived directly from the Social Institutions and Gender Index (SIGI) 2014 (OECD, 2014b). Within SIGI, the Restricted Civil Liberties sub-index indicates the constraints women face in accessing and participating in public and societal domains due to legal, procedural, or cultural barriers. This encompasses hindrances such as curtailed mobility for women, restricted access to public venues, barriers in political engagement, limits on freedom of association, and the need for male approval for journeys or passport acquisition. Limitations on women's active participation in the public sphere are significantly associated with wife abuse (Bunch, 1990). The data on domestic violence law were sourced from the Gender, Institutions and Development Database (GID-DB) 2014 (OECD, 2014a). The GID-DB is a component of the Social Institutions and Gender Index (SIGI) and comprises variables measuring the level of discrimination against women through laws, social norms, and practices across 160 countries (OECD, 2014a). The GID-DB includes a specific sub-index dedicated to monitoring infractions against women's physical integrity. It has been substantially demonstrated that legal safeguards are intrinsically tied to the incidence of wife abuse (Oppenlander, 1981). Domestic violence law variable evaluates the degree to which the legal system shields women from violence. All country-level variables are appended to the WVS individual-level dataset based on their respective countries.

The study sample consisted of 76,025 individual respondents from 51 countries, ranging from 1,000 respondents from Chile or Cyprus to 4,108 respondents from Canada. The average number of respondents per country was 1702.1 (SD = 756.7). Regarding sex, women are slightly more prevalent (female: 52.3%, male: 47.7%); the average age of respondents was 42.41 years (SD = 16.29). Educational attainment was evenly distributed: (low: 31.2%, middle: 36.1%, and high: 32.8%). A detailed list of participating countries is provided in the appendix.

### Measures

*Attitudes toward wife abuse*. The attitudes toward wife abuse were measured with a single item asking, “please tell me for each of the following statements whether you think it can always be justified, never be justified, or something in between for wife abuse.” The response options ranged from 1 (never justifiable) to 10 (always justifiable). The distribution was markedly positively skewed (skewness = 2.53, kurtosis = 9.17) because a significant number of respondents selected values of 1 or 2. To address this skewness in the 10-point scale regarding attitudes toward wife abuse, Thulin et al. (2022) transformed the scale into a dichotomous variable: 0 (never justifiable) and 1 (somewhat justifiable, by consolidating values 2–10). This study followed their procedure, employing it in multilevel binary logistic regression analyses.

#### Individual-level factors (level-1)

*Life Satisfaction.* Life satisfaction was measured using two items: “satisfaction with your life” and “satisfaction with the financial situation of the household.” The response option was a 10-point scale, ranging from 1 (completely dissatisfied) to 10 (completely satisfied). The scores from the two items were averaged, with higher scores indicating greater life satisfaction.

*Economic Hardship.* Economic hardship was measured using four items, all beginning with the prompt: “In the last 12 months, how often have you or your family:” Item examples included “gone without enough food to eat,” “gone without needed medicine or treatment,” “gone without a cash income,” and “gone without a safe shelter over your head.” Response options ranged from 1 (often) to 4 (never). These responses were then reverse coded and averaged. The Cronbach's alpha for economic hardship was 0.78.

*Lack of Trust.* Lack of trust was measured with three items. Respondents were prompted with: “I’d like to ask you how much you trust people from various groups. Could you tell me for each whether you trust people from this group completely, somewhat, not very much or not at all?” Items included “your family,” “your neighborhood,” and “people you know personally.” The response option ranged from 1 (trust completely) to 4 (do not trust at all). Prior studies combined the three items into a single index known as “in-group trust” (Crepaz et al., 2014; Delhey et al., 2011; Welzel & Delhey, 2015). This index has been used to examine the level of trust in one's close group. The current study followed their procedure and averaged three items. The higher the average, the higher the lack of trust. The Cronbach's alpha for lack of trust was 0.61.

*Gender Inequality.* Gender inequality was assessed using four items including: “if a mother works for pay, the children suffer,” “on the whole, men make better political leaders than women,” “university education is more important for a boy than for a girl,” and “generally, men make better business executives than women.” Response options ranged from 1 (strongly agree) to 4 (strongly disagree). The four items were reverse-coded and averaged. The higher the score, the greater the gender inequality. The Cronbach's alpha for gender inequality was 0.73.

*Neighborhood Risks.* Neighborhood violence was measured with eight items. Respondents were prompted with: “how frequently do the following things occur in your neighborhood?” Example items included “robberies” and “street violence and fights.” Response options were measured on a 4-point scale that ranged from 1 (very frequently) to 4 (not at all frequently). Items describing positive neighborhood experiences were reverse-coded for uniformity. All eight items were reverse-coded and averaged to serve as an indicator of neighborhood risks. This measure has been used in earlier studies (Thulin et al., 2022). The Cronbach's alpha for neighborhood risks stood at 0.87.

#### National-level factors (level-2)

*Democracy.* Democracy was measured using the EIU's Democracy Index from 2016 (EIU Digital Solutions, n.d.). This index was measured on a 0 to 10 scale and derives its score from 60 indicators, each rated as 0.0, 0.5, or 1.0. These indicators were organized into five categories: “electoral process and pluralism,” “civil liberties,” “functioning of government,” “political participation,” and “political culture.” Each category received a rating on a 0 to 10 scale, and the overall democracy index was the simple average of these five category ratings. A higher score on this scale represents greater democracy.

*Domestic Violence Law.* Domestic violence law was measured using the GID-DB 2014 (OECD, 2014a). This variable determines if the legal framework provides women with protection against domestic violence. The index used a rating of “0.00,” “0.25,” “0.50,” “0.75,” and “1.00.” The rating of “0.00” indicates “there is specific legislation in place to address domestic violence; the law is adequate overall, and there are no reported problems of implementation.” The rating of “0.25” indicates “there is specific legislation in place to address domestic violence; the law is adequate overall, but there are reported problems of implementation.” The rating of “0.50” indicates “there is specific legislation in place to address domestic violence, but the law is inadequate.” The rating of “0.75” indicates “there is no specific legislation in place to address domestic violence, but there is evidence of legislation being planned or drafted.” Lastly, the rating of “1.00” indicates “there is no legislation in place to address domestic violence”. In other words, the variable is coded on a scale from 0 to 1, with “0” indicating strong legal protection against domestic violence and “1” signifying a complete lack of such protection.

*Restricted Civil Liberties.* The Restricted Civil Liberties was measured using the Restricted Civil Liberties item from the SIGI Index 2014 (OECD, 2014b). The Restricted Civil Liberties is a sub-index that identifies laws and practices that limit women's participation and voice in public and social domains. The variable is coded on a scale from 0, representing no or minimal restricted civil liberties, to 1, indicating extremely high restricted civil liberties.

#### Covariates

Socio-demographic variables were included as covariates which included personal characteristics such as age (in years), sex (female =1 vs. male = 0), and educational level. The respondents’ educational level was categorized into three groups based on the International Standard Classification of Education (ISCED) categories (UNESCO Institute for Statistics, 2012). “Low” education indicates early childhood education, primary education, and lower secondary education; “medium” education indicates upper secondary education and post-secondary non-tertiary education; and “high” education indicates short-cycle tertiary education, Bachelor's degree, Master's degree, and Doctoral degree or equivalent tertiary education level.

#### Data analysis

A multilevel binary logistic model was employed to examine the associations between individual-level characteristics (level-1 variables) nested within country-level characteristics (level-2 variables) in justifying wife abuse. Table 2 presents the unconditional model and three hierarchical random-intercept and fixed-slope multilevel models. Model 0 estimates the proportion of the variance in respondents’ attitudes toward wife abuse justification that can be attributed to between-country differences. Model 1 examines the association between individual characteristics (controlling for covariates) and the attitudes toward wife abuse. Model 2 includes country-level variables to model 1, demonstrating the influence of country-level variables on the likelihood of positive attitudes toward wife abuse. Model 3 includes interaction terms between sex (level-1) and democracy, domestic violence law, and restricted civil liberties (level-2) variables. All statistical analyses were conducted using Stata software (version 15). The multilevel binary logistic model used in this study is represented as follows:

$$Level\;1:\;\;logit(P(y_{ij}=1))=\beta_{0j}+X_{ij}\beta +Z_{ij}\delta +\varepsilon_{ij},$$

$$\begin{array}{rl} Level\;2:\;\;\beta_{0j} & =\gamma_{00}+u_{0j},\;u_{0j}\;∼\;N(0,\tau^{2})\\ \beta & =\gamma \\ \delta & =\alpha \end{array},$$

$$Combined\;Model:logit(P(y_{ij}=1))=\gamma_{00}+X_{ij}\gamma +Z_{ij}\alpha +u_{0j},$$

$$\begin{array}{rl} & *i:Individual,\;j:\;Country,\;X_{ij}:Matrix\;of\;fixed\;effect\;predictors,\\ & Z_{ij}:Matrix\;of\;interaction\;terms. \end{array}$$

## Results

### Descriptive Statistics

Table 1 displays the descriptive statistics of the variables and the associations between individual-level variables and the positive attitudes toward wife abuse across 51 countries, using the t/chi-square test of independence. The results indicated that following variables—level-1 predictors: life satisfaction (*t* = 19.97; *p* < .001), neighborhood risks (*t* = −24.71; *p* < .001), lack of trust (*t* = −9.27; *p* < .001), economic hardship (*t* = −35.98; *p* < .001), and gender inequality (*t* = −42.7; *p* < .001) and control variables at level-1: age (*t* = 19.27; *p* < .001), sex (chi-square = 383.25; *p* < .001), and level of education (chi-square = 270.51; *p* < .001)—were statistically significant in differences between individuals who had positive attitudes toward wife abuse and individuals who do not have positive attitudes toward wife abuse.

**Table 1.** Descriptive Statistics.

	Total (*n* = 76,025)			*χ*^2^/*t*-value
Variables	*N*	Mean (SD)/%	Min-Max	
Demographics				
Age	75,902	42.41 (16.29)	16–103	19.27***
Sex	75,970			383.25***
Male	36,264	47.73%		
Female	39,706	52.27%		
Education	75,452			270.51***
High	24,714	32.75%		
Middle	27,230	36.09%		
Low	23,508	31.16%		
DV (level-1)				
Wife abuse	75,280			
Justifiable	20,151	26.77%		
Not justifiable	55,129	73.23%		
IV (level-1)				
Life satisfaction	75,883	6.53 (2.11)	1–10	19.97***
Neighborhood risks	75,723	1.82 (0.68)	1–4	−24.71***
Lack of trust	75,932	1.87 (0.56)	1–4	−9.27***
Economic hardship	75,899	1.62 (0.71)	1–4	−35.98***
Gender inequality	75,815	2.39 (0.63)	1–4	−42.7***
IV (level-2)				
Democracy	76,025	5.92 (1.87)	1.9–9.3	
Domestic violence Law	76,025			
0	21,419	28.17%		
0.25	22,141	29.12%		
0.5	18,676	24.57%		
0.75	7,471	9.83%		
1	6,318	8.31%		
Restricted civil liberties	76,025	0.39 (0.24)	0–1	

*Note*. Low education indicates elementary and pre-secondary education (early childhood education, primary education, and lower secondary education), middle education indicates secondary and post-secondary (upper secondary education and post-secondary non-tertiary education), and high education indicates college or above (short-cycle tertiary education, Bachelor's degree or equivalent tertiary education level, Master's degree or equivalent tertiary education level, and doctoral degree or equivalent tertiary education level).

Domestic violence law:

0: The legal framework protects women from domestic violence, without any legal exceptions and in a comprehensive approach.

0.25: The legal framework protects women from domestic violence, without any legal exceptions. However, the approach is not comprehensive.

0.5: The legal framework protects women from domestic violence. However, some legal exceptions occur.

0.75: The legal framework protects women from some forms of domestic violence but not all.

1: The legal framework does not protect women from domestic violence.

Table 2 presents the multilevel logistic regression model. In Model 0, the intra-class correlation (ICC) was 0.21, suggesting that 21% of the variance in the positive attitudes toward wife abuse is attributable to differences between countries. The model also highlights that the unconditional probability of positive attitudes toward wife abuse, calculated as [0.32/(1 + 0.32)], stands at 24% across 51 countries. Model 1 results demonstrated the association between individual-level variables and positive attitudes toward wife abuse while controlling for the covariates (level-1). The intra-class correlation (ICC) for Model 1 decreased to 20% (ICC = 0.2) from 21% (ICC = 0.21) in the unconditional model, indicating a variance reduction of 1% in the positive attitudes toward wife abuse by adding level-1 predictors and controlling for covariates. The findings showed that gender inequality was significantly associated with positive attitudes toward wife abuse. In particular, individuals with tendencies toward gender inequality were significantly more likely to have positive attitudes toward the justification of wife abuse (OR = 1.44; 95% CI: 1.21–1.72; *p* < .001). Regarding covariates, older individuals (age, OR: 0.74; 95% CI: 0.99–1.00; *p* < .001), females (OR: 0.73; 95% CI: 0.68–0.8; *p* < .01), and individuals with higher educational attainment (OR: 0.88; 95% CI: 0.83–0.94; *p* < .001) had significantly lower odds of the positive attitudes toward wife abuse.

**Table 2.** Multilevel Logistic Regression (Odds Ratio).

	Attitudes toward wife abuse							
	Model 0^a^		Model 1^b^		Model 2^c^		Model 3^d^	
	Estimates (robust SE)	95% CI	Estimates (robust SE)	95% CI	Estimates (robust SE)	95% CI	Estimates (robust SE)	95% CI
Intercept	0.32*** (0.04)	0.25–0.42	0.92 (0.73)	0.19–40.4	0.52 (0.43)	0.1–2.68	0.52 (0.44)	0.1–2.68
Level-1 variable								
*Covariates (level-1)*								
Age			0.99** (0.00)	0.99–0.1	0.99** (0.00)	0.99–0.1	0.99** (0.00)	0.99–0.1
Female			0.74*** (0.03)	0.68–0.8	0.74*** (0.03)	0.68–0.8	0.73*** (0.03)	0.68–0.79
Education level			0.88*** (0.03)	0.83–0.94	0.88*** (0.03)	0.83–0.94	0.88*** (0.03)	0.83–0.94
*Level-1 variable*								
Life satisfaction			1.11 (0.20)	0.77–1.59	1.01 (0.18)	0.71–1.44	1.01 (0.18)	0.71–1.44
Neighborhood risks			0.75 (0.44)	0.24–2.35	0.54 (0.27)	0.2–1.46	0.53 (0.27)	0.2–1.46
Lack of trust			2.16 (1.26)	0.69–6.75	1.93 (1.25)	0.54–6.86	1.93 (1.25)	0.54–6.84
Economic hardship			0.52 (0.22)	0.22–1.21	0.91 (0.42)	0.37–2.25	0.91 (0.42)	0.37–2.25
Gender inequality			1.44*** (0.13)	1.21–1.72	1.44*** (0.13)	1.21–1.72	1.44*** (0.13)	1.21–1.72
*Level-2 variable*								
Democracy					0.79* (0.09)	0.64–0.98	0.82* (0.08)	0.67–1.0
Domestic violence law					0.61 (0.32)	0.22–1.72	0.74 (0.36)	0.29–1.19
Restricted Civil liberties					1.17 (0.70)	0.37–3.75	1.15 (0.62)	0.4–3.3
*Interaction effects*								
Democracy × Female							0.93 (0.04)	0.87–1.01
Domestic Violence Law × Female							0.68** (0.10)	0.51–0.9
Restricted Civil Liberties × Female							1.04 (0.17)	0.75–1.43
Between country Variance	0.87 (0.17)	0.59–1.28	0.8 (0.17)	0.53–1.23	0.7 (0.12)	0.5–0.97	0.69 (0.12)	0.5–0.97
ICC (intra-class correlation)	0.21 (0.03)	0.15–0.28	0.2 (0.03)	0.14–0.27	0.17 (0.02)	0.13–0.23	0.17 (0.02)	0.13–0.23
Wald’s test			152.01***		169.5***		204.66***	
Observations	75,280		74,428		74,428		74,428	

*Note*. Exponentiated coefficients; robust standard errors in parentheses; *p < .05, **p < .01, ***p < .001.

Model 1: LR test vs. logistic model: chibar2(01) = 9748.8; Prob ≥ chibar2 = 0.000.

a Model 0 (unconditional model) estimates the variance in attitudes toward wife abuse justification attributable to between-country differences.

b Model 1 (level-1) estimates individual characteristics, controlling for covariates.

c Model 2 (level-1 and level-2) includes country-level variables to Model 1 to assess their influence.

d Model 3 (level-1 and level-2 and interaction terms) includes interaction terms between sex and democracy, domestic violence law, and restricted civil liberties.

Model 2 builds upon Model 1 by including country-level variables. ICC for model 2 decreased to 17% (ICC: 0.17) from 21% (ICC: 0.21) in the unconditional model. The overall variance reduction in the positive attitude toward wife abuse was 21% points from the unconditional model, with a 3% point reduction attributed to level-2 predictors and a 1% point reduction to level-1 predictors. The findings suggest that higher levels of democracy were associated with lower odds of positive attitudes toward wife abuse. In countries with greater democratic practices, the odds of positive attitudes toward wife abuse were significantly lower (OR = 0.79; 95% CI: 0.64–0.98; *p* < .05). This result is consistent with previous studies that found higher levels of democratic values were statistically significantly associated with lower positive attitudes toward wife abuse at the national level (Sardinha & Nájera Catalán, 2018).

Model 3 includes interaction terms between sex (level-1) and democracy, domestic violence law, and restricted civil liberties (level-2) variables. The results showed significant interactions between sex and domestic violence law. This indicates that the effects of domestic violence legislation on the positive attitudes toward wife abuse vary by sex. In countries with weaker protections under domestic violence law, women are also less likely than men to justify attitudes toward wife abuse (OR: 0.68; 95% CI: 0.51–0.90; *p* < .01).

**Figure 1.**
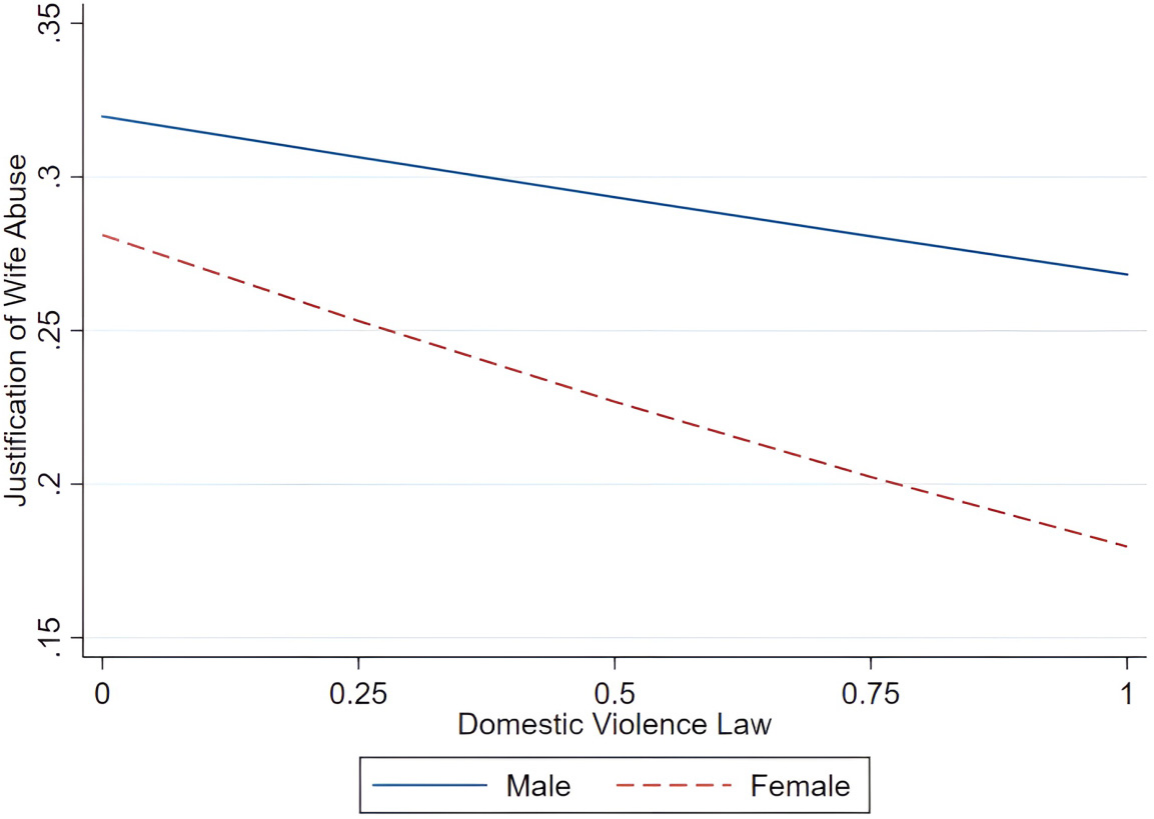
Interactions between domestic violence law, sex, and attitudes toward wife abuse.

Figure 1 shows that predicted attitudes toward wife abuse decreased significantly with the lack of domestic violence law, particularly among women. This finding suggests that women's attitudes appear to be relatively less supportive of wife abuse than men's attitudes in environments with weaker legal protections against domestic violence. These results underscore the importance of considering both individual characteristics and national context, as well as their complex interplay when examining positive attitudes toward wife abuse.

## Discussion

To comprehensively understand the factors associated with the positive attitudes toward IPVAW on a global scale, guided by the ecological framework, this study used MLM combining multiple data sources (global datasets from WVS, the OECD database, and EIU digital solutions) of 76,025 individuals across 51 countries. Our study focused on two levels of risk factors: the individually experienced risk (level-1) and national level risk (level-2). Regarding level-1 variables (individual, interpersonal, and neighborhood domains), gender inequality was found to be a significant risk factor, while the level-2 variable (national domain) showed that democracy was the unique factor that decreased the level of attitudes justifying wife abuse. An interaction effect was significant between sex and domestic violence law in association with positive attitudes toward IPVAW.

Regarding individually experienced risk factors (level-1 risk), gender inequality was found to increase positive attitudes toward IPVAW. Although societies across the globe have struggled to improve women's rights, the perception of gender inequality still lingers, increasing the risk of holding positive attitudes toward IPVAW. Supported by prior research (Sánchez-Prada et al., 2023), gender inequality shapes one's attitudes toward IPVAW. Since the perception of gender inequality is challenging to improve within a short period, a consistent long-term endeavor throughout the globe may be necessary. On a global scale, for example, UN Women (UN Women, 2024), which is an organization at the United Nations, focuses on improving women's rights across the globe by empowering employment, increasing leadership and political participation, and ending violence against women. Also, multiple efforts on the individual, family, school, and society are imperative to reduce perceptions of gender inequality to reduce positive attitudes toward IPVAW.

The national level factors showed that democracy plays a critical role in reducing positive attitudes toward IPVAW. As the core value of democracy seeks peace and support of human rights, individuals are discouraged from pursuing aggression and violence under a democratic system (U.S. Department of State, 2024). Attitudes toward IPVAW may be shaped in a relatively long-term period within a sociocultural atmosphere. Although the process of instilling the value of democracy takes generations, the systematic approach for promoting and encouraging democratic values has successfully discouraged individuals from having positive attitudes toward IPVAW. Consistent with Schwarzmantel (2011), rational thought processes can be cultivated in a democratic society that can reject the attitude toward IPVAW. Most importantly, democracy enables freedom of expression, allowing women to speak out against IPVAW. As a result, under circumstances where violence against women is strongly discouraged and where there is freedom to express the unjust of violence against women, attitudes toward IPVAW can decrease. Hence, national endeavors to promote democratic values through multiple channels such as education, politics, and media shed light on reducing attitudes that justify IPVAW.

Although there was no significant main effect of domestic violence law and positive attitudes toward IPVAW, unexpected findings were revealed in the interaction analysis.

In general, the existence of domestic violence laws decreases the actual rate of IPVAW (Modi et al., 2014). However, our findings showed that in countries with weaker protections under domestic violence law, women were less likely to condone IPVAW compared to men. The findings may seem paradoxical at first glance. However, considering that our study focused not on the actual but the attitudes condoning IPVAW, the presence of domestic violence legislation may not necessarily reduce attitudes condoning IPVAW, particularly among women. Hypothetically, legislation to punish batterers and protect victims of IPVAW is not needed in societies where incidents of IPVAW do not exist (Jutting et al., 2008). A stronger legal framework is necessary when the actual IPVAW is prevalent in society, which in turn, aims to prevent IVPAW. Hence, the attitudes toward IPVAW may not directly be associated with domestic violence laws.

Instead, it should be emphasized that the focus of our study was the attitudes toward IPVAW, and our findings presented critical clues to find channels to link domestic violence laws to reduce the positive attitudes toward IPVAW. As prior research highlights, countries with stronger enforcement of rights and legal systems that do not tolerate IPVAW may decrease the risk of IPVAW, particularly among women (Barajas-Sandoval et al., 2023). However, as it was found in our study, simply enacting more legislation may not be the solution to improving responses to IPVAW (Walklate et al., 2018); instead, the process, adequate resources, and proper implementation of these laws matter. Despite the existence of strong domestic violence laws, as reflected in our findings, the population's awareness of the laws may be critical to reducing the positive attitudes toward IPVAW (Song et al., 2017). In other words, merely enacting domestic violence laws may not change positive attitudes toward IPVAW, particularly among women. Instead, proper implementation, resources, and public awareness of these laws are crucial. From a prevention approach, the deterrent effects of the domestic violence laws would not fully work as expected unless the large population is aware of the existence of the domestic violence laws. Therefore, educating the general population—especially women—about existing laws may further encourage them to resist to positive attitudes toward IPVAW. For example, a large number of people in the United States (U.S.) attempt to resolve IPVAW problems through informal channels instead of seeking legal assistance (Lee & Backes, 2018). Countries with fewer resources and law protections will be unlikely to distribute multiple channels to dissipate the information of the existence of domestic violence laws that can protect women from IPVAW.

The study's limitations are as follows. First, certain countries, including Andorra, Taiwan, Macau, Puerto Rico, and Vietnam, were not included in the analysis because the SIGI and GIB-DB datasets do not cover these countries. Additionally, China was excluded due to the absence of the neighborhood disadvantage variable in the WVS. Second, this study used a cross-sectional dataset, so causality among the variables should not be inferred. Third, although the ecological framework highlights the nested effects of multiple domains, our study design emphasized the individual experience (level-1) and national structure (level-2) factors associated with attitudes toward IPVAW. Fourth, although we hypothesized that women with low life satisfaction are likely to experience more abuse, the opposite relationship could be possible. In other words, women exposed to IPVAW are likely to experience lower life satisfaction or show a reciprocal association. Therefore, we encourage future studies to further clarify the possibility of endogeneity between the variables. Fifth, although we were not able to examine this in our analysis due to the restriction of the data, a possible third variable, such as exposure to childhood abuse, could partially explain the correlation between gender inequality and attitudes toward spousal abuse. As traumatic childhood experiences negatively shape one's worldview, they might influence individuals to justify spousal abuse or adopt gender-unequal attitudes.

## Conclusion and Implications

This study explored individual- and national-level factors associated with attitudes toward IPVAW using an MLM study design and a new synthetic dataset aimed at overcoming limitations from previous studies. The study makes a new contribution to the growing literature on the application of the ecological framework to understand and address IPVAW across the globe. Our findings suggest that improving individual perceptions of gender inequality and emphasizing the values of democracy are particularly critical to reducing positive attitudes toward IPVAW. Furthermore, countries with domestic violence laws may be particularly important for women, as they are the primary victims of these incidents. Hence, along with enacting stronger and protective legislation against IPVAW, educating women about their rights and existing domestic violence laws may be a key mechanism to reduce attitudes condoning IPVAW. Based on the findings of this study, clinicians, policymakers, legislators, and researchers should consider multiple sources of factors associated with attitudes toward IPVAW across ecological factors and apply them to multiple settings. Policymakers, in particular, should consider funding programs that teach gender equality in education systems. Additionally, the findings of this study support efforts to strengthen democracies, such as increasing civic engagement, free media, and political participation by women, which will likely reduce justifications for IPVAW. Policies that penalize domestic violence are also important to implement to reduce IPVAW. By implementing policies that focus on education, legal enforcement, democracy promotion, public awareness, and socioeconomic interventions, governments can ameliorate positive attitudes toward IPVAW.

## Appendix

**Table A1.** List of Variables

Variable	Description	Scale
Wife abuse	Justifiable: For a man to beat his wife	No (Not justifiable)
		Yes (Somewhat justifiable)
Sex	Sex	Male
		Female
Age	Age	
Education	Highest educational level: Respondent (ISCED 2011)	Low: Elementary and pre-secondary education (early childhood education, primary education and lower secondary education)
		Medium: Secondary and post-secondary (Upper Secondary Education and Post-secondary non-Tertiary Education
		High: College or above (Short-cycle tertiary education, Bachelor's degree or equivalent tertiary education level, Master's degree or equivalent tertiary education level, and Doctoral degree or equivalent tertiary education level).
Life satisfaction	Satisfaction with your life	1 = dissatisfied to 10 = Satisfied
	Satisfaction with financial situation of household	
Neighborhood risks	Frequency in your neighborhood: Robberies	1: Not at all frequently to 4: Very Frequently
	Frequency in your neighborhood: Alcohol consumed in the streets	
	Frequency in your neighborhood: Police or military interfere with people's private life	
	Frequency in your neighborhood: Racist behavior	
	Frequency in your neighborhood: Drug sale in streets	
	Frequency in your neighborhood: Street violence and fights	
	Frequency in your neighborhood: Sexual harassment	
Lack of trust	How much you trust: Your family	1: Trust completely to 4: Do not trust at all
	Trust: Your neighborhood	
	Trust: People you know personally	
Economic hardship	Frequency you/family (last 12 month): Gone without enough food to eat	1: Never to 4: Often
	Gone without needed medicine or treatment that you needed	
	Gone without a cash income	
	Gone without a safe shelter over your head	
Gender inequality	Pre-school child suffers with working mother	1: Strongly disagree to 4: Strongly agree
	Men make better political leaders than women do	
	University is more important for a boy than for a girl	
	Men make better business executives than women do	
Democracy	Economist Intelligence Unit's Democracy Index (EIU)	From 0 to 10 scale (A higher score on this scale represents greater democracy)
Domestic violence law	The level of discrimination against women through laws, derived from the Gender, Institutions and Development Database (GID-DB)	0: The legal framework protects women from domestic violence, without any legal exceptions and in a comprehensive approach;
		.25: The legal framework protects women from domestic violence, without any legal exceptions. However, the approach is not comprehensive;
		.5: The legal framework protects women from domestic violence. However, some legal exceptions occur.
		.75: The legal framework protects women from some forms of domestic violence but not all.
		1: The legal framework does not protect women from domestic violence.
Restricted civil liberties	The constraints women face in accessing and participating in public and societal domains due to legal, procedural, or cultural barriers, derived from the Social Institutions and Gender Index (SIGI)	From 0, representing no or minimal restrictions on civil liberties, to 1, indicating extremely high restrictions on civil liberties.
